# Therapeutic Oligonucleotides: An Outlook on Chemical Strategies to Improve Endosomal Trafficking

**DOI:** 10.3390/cells12182253

**Published:** 2023-09-11

**Authors:** Priyanka Mangla, Quentin Vicentini, Annabelle Biscans

**Affiliations:** 1 Oligonucleotide Discovery, Discovery Sciences Research and Development, AstraZeneca, 431 38 Gothenburg, Sweden; priyanka.mangla@astrazeneca.com (P.M.); quentin.vicentini1@astrazeneca.com (Q.V.); 2 Department of Laboratory Medicine, Clinical Research Centre, Karolinska Institute, 141 57 Stockholm, Sweden

**Keywords:** oligonucleotide therapeutics, antisense oligonucleotides (ASOs), siRNA, oligonucleotide delivery, endosomal escape, endosomolytic agents, oligonucleotide conjugates, linker chemistry, non-cleavable linkers, cleavable linkers, pH-sensitive linkers

## Abstract

The potential of oligonucleotide therapeutics is undeniable as more than 15 drugs have been approved to treat various diseases in the liver, central nervous system (CNS), and muscles. However, achieving effective delivery of oligonucleotide therapeutics to specific tissues still remains a major challenge, limiting their widespread use. Chemical modifications play a crucial role to overcome biological barriers to enable efficient oligonucleotide delivery to the tissues/cells of interest. They provide oligonucleotide metabolic stability and confer favourable pharmacokinetic/pharmacodynamic properties. This review focuses on the various chemical approaches implicated in mitigating the delivery problem of oligonucleotides and their limitations. It highlights the importance of linkers in designing oligonucleotide conjugates and discusses their potential role in escaping the endosomal barrier, a bottleneck in the development of oligonucleotide therapeutics.

## 1. Introduction

**Figure 1:**
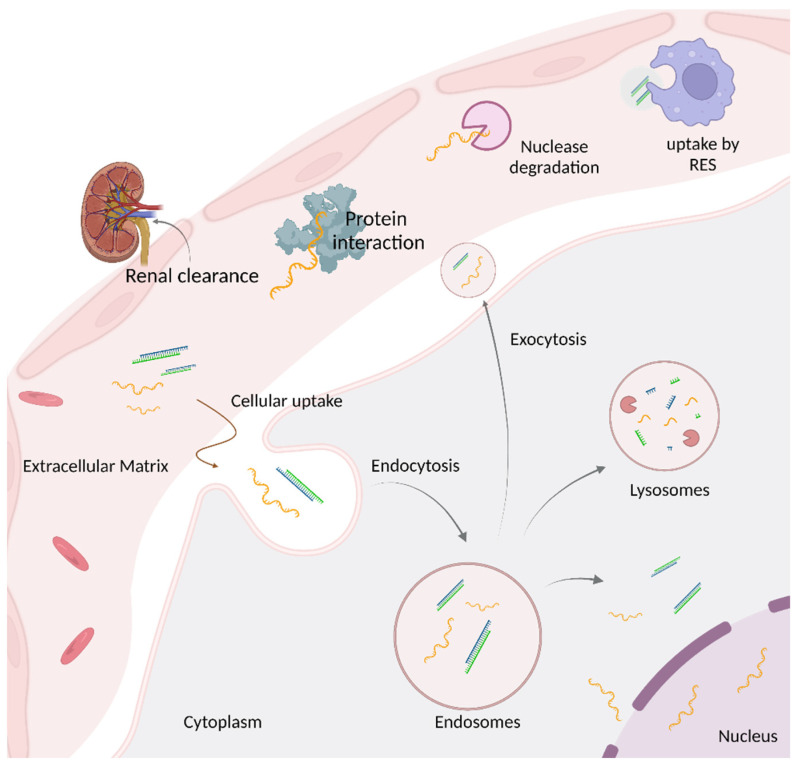
Biological barriers preventing therapeutic activity of oligonucleotides. Systemically administered oligonucleotides encounter nuclease degradation, RES phagocyte uptake, protein interaction, and renal excretion before reaching the specific tissue/cell of interest. On reaching the tissue/cell, intracellular uptake occurs via endocytosis. Most oligonucleotides either get trapped in endosomes and further transferred to lysosomes where they encounter enzymatic degradation or are recycled back into extracellular matrix via exocytosis. Only 1–2% of administered oligonucleotides escape the endosomes to reach the target in cytosol or nucleus. Created with BioRender.com, AO25FLAM37.

Oligonucleotide therapeutics are synthetically modified nucleic acids that can modulate gene expression via a range of processes, including RNA interference, target degradation by RNase H-mediated cleavage, splicing modulation, gene editing, and gene activation [1,2]. In contrast to conventional drugs, oligonucleotides have the potential to target traditionally undruggable disease-causing genes and patient-specific sequences that are conducive to rare diseases. However, to attain clinical success, they need to overcome multiple biological barriers to be efficiently delivered into the targeted cells/tissues and reach their target (Figure 1) [3,4]. Chemical modifications of oligonucleotides have proven to be crucial to improve oligonucleotide drug properties. The diligent efforts on oligonucleotide chemistry for more than half a century have resulted in increasing oligonucleotide on-target activity and metabolic stability while decreasing off-target effects and immunogenicity [5,6,7,8]. As of today, there are more than 15 FDA-approved oligonucleotide-based drugs mainly of small interfering RNAs (siRNAs) and antisense oligonucleotide (ASO) classes; latter including, in particular, splice switching oligonucleotides (SSOs), and encompassing either phosphorothioate (PS) oligonucleotides or phosphorodiamidate morpholino oligomers (PMOs), to target various disease-causing genes in the liver, CNS, and muscles [9,10,11].

Local delivery of oligonucleotides to the eye, spinal cord, and brain has been successfully achieved via intravitreal (IVT) and intrathecal (IT) administration, respectively [12,13]. However, systemic administration of oligonucleotides has been less successful due to poor tissue distribution and uptake (Figure 1). While both lipid nanoparticles and *N*-acetylgalactosamine (GalNAc) conjugates are clinically validated and approved delivery strategies for liver targets, efficient delivery of oligonucleotides beyond the liver is a fundamental obstacle preventing their clinical utility [14,15,16,17,18,19]. Alternative approaches such as lipid conjugation, exosome loading, enveloped virus, spherical nucleic acids (SNAs), DNA cages, and smart materials show great promise to improve oligonucleotide delivery into specific tissues [1,20,21,22,23,24,25,26].

After reaching the tissue of interest, oligonucleotides, whether non-conjugated, conjugated, or formulated, enter the cell through endocytosis [27]. As oligonucleotides are polyanionic macromolecules, escaping the endosomal lipid bilayers to reach their intracellular targets represents a major obstacle, limiting the development of safe and efficient drugs. Recently, it has been reported using quantitative NanoSIMS microscopy that only 1–2% of *N*-acetylgalactosamine (GalNAc)-conjugated ASOs escape endosomes to engage with the target in vivo [20,28,29]. However, the mechanistic understanding of how the oligonucleotides escape from endosomes is still not understood. Various hypotheses have been proposed in the literature: 1. The repetitive, spontaneous, and short-lived, small breaches that occur in the endosomal lipid bilayer can allow the escape of oligonucleotides into cytosol. 2. Oligonucleotides can escape through a temporary breach generated during the fusion events between endosomes, multivesicular bodies (MVBs), and lysosomes. 3. Oligonucleotides may escape via retro-transport from the Golgi [20,29,30]. Understanding the spontaneous mechanism of endosomal escape will be the key to improve oligonucleotide endosomal release and thus enhance oligonucleotide activity and the therapeutic window.

As demonstrated from clinical successes of oligonucleotide therapeutics, <1% of endosomal escape is sufficient to achieve robust and long-lasting activity in the liver when using GalNAc conjugation. A single subcutaneous administration of GalNAc-conjugated siRNAs can induce a clinical benefit that lasts up to 6–12 months in humans [18,19]. However, the low level of endosomal release is insufficient when targeting extra-hepatic tissues. In addition to limited endosomal escape, most oligonucleotides are degraded by lysosomal enzymes or recycled back to plasma membrane via exocytosis before they can escape the endosomes, further limiting the amount of oligonucleotides available to reach the target [31]. Therefore, addressing the endosomal escape problem is required to efficiently treat widespread diseases and as Steven Dowdy mentioned, “it will not be easy, otherwise it would have already been solved” [20].

The aim of this review is to provide a comprehensive summary of chemical advances made in oligonucleotide delivery and in developing non-viral systems that can facilitate the endosomal escape. It highlights the importance of linker chemistry in designing oligonucleotide conjugates and discusses their potential role in escaping the endosomal barrier.

## 2. Chemical Modifications to Improve Oligonucleotide Drug Properties

Chemical modifications are required to use oligonucleotides as therapeutics [1,32]. They are essential to improve oligonucleotide stability and bioavailability and enhance target binding affinity. For decades, many chemical entities have been explored and incorporated into oligonucleotides, mainly on the backbone and the ribose, but also on the nucleobase [8,33]. Each modification confers oligonucleotide-specific properties and may be incompatible with certain classes of oligonucleotide therapeutics as they can alter their mode of action. Therefore, depending on the oligonucleotide class (e.g., ASO or siRNAs) incorporation of well-designed chemical modifications is primordial to maintain activity (Table 1). In this section, the most common chemical modifications used in approved and clinical ASOs and siRNAs will be discussed as the scope of modifications has been thoroughly described in other reviews [1,9].

### 2.1. Chemical Modifications in ASOs

**Figure 2:**
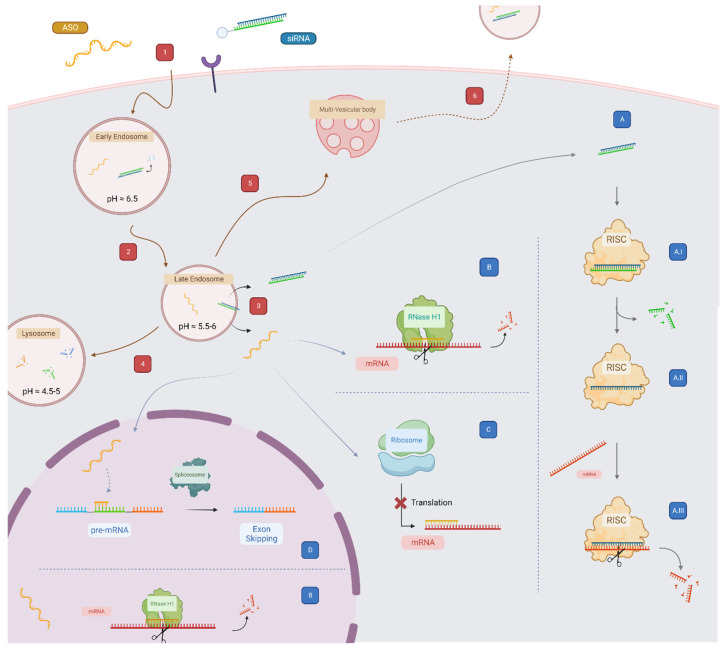
Summary of trafficking and mode of action of ASOs and conjugated siRNAs. (**1**) Oligonucleotides are first internalized into the endosomal pathway by gymnotic uptake or by receptor-mediated uptake, (**2**) in early endosomes, dissociation with the targeting moiety or ligand is initiated, and (**3**) maturation of the early endosome leads to late endosomes where oligonucleotides are found to escape in the cytosol. Late endosomes can either mature to (**4**) lysosomes, where most of the material is degraded by the low-pH and degradation enzymes, or (**5**) to Multivesicular bodies where they are (**6**) exported to the external environment. In the cytoplasm, (**A.I**) siRNAs are loaded into RISC, where (**A.II**) the passenger strand is degraded and the guide strand selected. After target recognition by the guide strand (**A.III**), the mRNA transcript is cleaved and degraded. For ASOs, they can either (**B**) degrade the target mRNA through the RNAse H1 enzyme or (**C**) block the translation machinery of the transcript. In the nucleus, ASOs can interfere with (**D**) the splicing machinery and generate a modified mature mRNA or (**B**) directly downregulate the target through RNAse H1. Created with BioRender.com, VP25FLPEXQ.

Since the first proof of concept, many different chemistries and the ASO’s mode of action have been thoroughly studied [34]. ASOs are short (about 20 mer long) and single-stranded synthetic nucleic acids. They have been successfully used for modulating the expression of transcripts, either by direct cleavage of the target RNA through the RNase H1 endonuclease, or by acting as steric blockers by interfering with the translation machinery of the transcript (Figure 2) [35]. 

**Figure 3:**
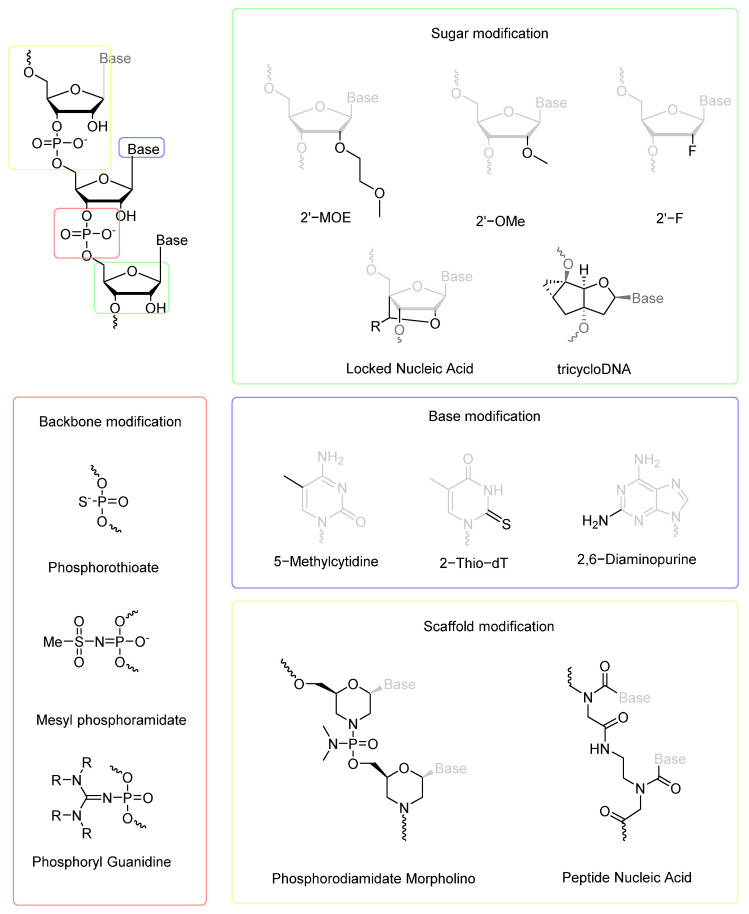
Examples of chemical modifications used to improve the therapeutic activity of oligonucleotides.

A range of modifications have been introduced to enhance ASO stability, efficacy, and delivery. The most common modifications consist of (i) substituting the phosphodiester (PO) backbone with a phosphorothioate (PS); (ii) modifying the 2′ position of the ribose; (iii) incorporating 5-methyl group on pyrimidine nucleobases; or (iv) introducing an artificial biopolymer scaffold, such as a PMO or peptide-nucleic acid (PNA) (Figure 3).

ASOs have greatly benefited from the PS backbone modification as made by Eckstein in 1966 that simply replaces one of the non-bridging oxygen atoms of the PO backbone with sulphur [36]. It confers the ASO a better serum stability towards endogenous nucleases, but also improves its pharmacokinetic (PK) properties [4,37]. PS-ASOs have a longer circulation time in vivo that correlates with an enhanced binding to plasma proteins [38,39]. This “simple” modification leads to a productive cellular uptake that is not observed with the canonical PO and thus ignited the clinical potential of ASOs [40]. The PS backbone also generates a broader interactome between the ASO and membrane-bound proteins and, depending of the protein-ASO pair, initiates different endocytotic pathways [41]. The latest work suggests a dynamic covalent exchange between the PS and the thiols/disulfides from proteins, resulting in a “thiol-mediated uptake” [42]. The PS modification led eventually to the first generation of antisense drugs in 1998, with the FDA approval of Fomivirsen to treat cytomegalovirus retinitis in immunodeficient patients (today withdrawn from the market) [43]. Additional backbone chemistries are also explored (Figure 3), with for instance, phosphoryl guanidine (PN) [44,45], a charge-neutral backbone analogue that shows improved resistance towards nucleases, or mesyl phosphoramidate that shows advantages in terms of binding affinity to the target RNA, nuclease resistance and enhanced RNase H1 cleavage when used in the gapmer context [46,47,48].

While the PS modification improves ASO stability and bioavailability, it also reduces the affinity for the target RNA. Therefore, incorporation of additional modifications is likely required to enhance ASO activity. 2′-modifications (e.g., 2′-*O*-methyl (2′-OMe), 2′-*O*-methoxy-ethyl (2′-MOE), and 2′-fluoro (2′-F) modifications) or constrained ribose and bridged nucleic acid modifications (e.g., locked nucleic acid (LNA) and tricyclo-DNA (tcDNA) modifications) enable superior binding affinity to RNA, ASO nuclease resistance, and can have a significant impact on ASO distribution. Furthermore, introduction of the 5-methyl group on cytosine reduces the immunostimulatory profile of certain DNA oligonucleotides and enhances nuclease stability (Figure 3) [49,50]. While the 5-methylcytosine is, to this day, the only base modification found in the clinic, it is worth nothing that many other chemistries are explored, such as 2-thio dT or 2,6-diaminopurine [51,52]. The combination of a PS backbone, 2′-*O*-alkyl modifications, and 5-methylcytidine led to Nusinersen and Mipomersen, two approved ASOs for the treatment of spinal muscular atrophy (SMA) and homozygous familial hypercholesterolemia, respectively [53,54].

Dramatic chemical changes in ASO scaffolds have also been developed (e.g., PMO and PNA), increasing ASO stability and modulating affinity for the target RNA. These non-charged backbone ASOs allow specific RNA targeting in an increasing number of clinical therapeutic areas. Eteplirsen is the first example of an FDA-approved PMO ASO for the treatment of Duchenne muscular dystrophy (DMD) [55]. Golodirsen, Viltolarsen, and Casimersen have followed, targeting different mutations in the DMD gene [56,57,58].

### 2.2. Chemical Modifications in siRNAs

siRNAs are short double-stranded RNAs (20 to 25-mer RNA duplexes) naturally present in eukaryotic cells [59]. Their primary role is to regulate gene expression via the RNA interference (RNAi) machinery [60]. Extensive studies on their mode of action (Figure 2) enable siRNA design optimization to utilize them as powerful lab tools and drugs for degradation of specific disease-causing mRNA.

As with ASOs, to be effective as a drug, siRNAs also require chemical stabilization. Chemical modifications that replace 2′-hydroxyl of the ribose and modify terminal nucleotide linkages are needed to maximize their in vivo activity [32,61,62].

The chemical modification of siRNAs is more challenging due to their double-stranded nature and the necessity to interact with the RNAi machinery. Bulky substituents on the 2′-position of the ribose are generally not well tolerated, and chemists combine smaller modifications like 2′-F and 2′-Ome, which do not prevent RNA-induced silencing complex (RISC) assembly and function [63]. Both modifications are approved in clinic and have demonstrated efficiency to enhance siRNA stability, increase affinity to the targeted mRNA, induce the duration of the effect, and reduce immunogenicity [9].

In addition to 2′-ribose modifications, further nuclease stability is primordial to efficiently deliver siRNAs to tissues, which can be achieved by incorporating PS linkages. High PS modification content results in a decrease in RNAi activity [64,65]. Therefore, in combination with 2′-OMe/2′-F modifications, clinically approved siRNAs only contain a maximum of two PS modifications at the strands’ termini [16,17,18].

In contrast to PS-ASOs, chemical modifications are not sufficient to significantly increase the cellular uptake of siRNAs. Therefore, formulation and conjugation strategies are required for an efficient delivery into tissues/cells of interest. As of today, *N*-acetylgalactosamine (GalNAc) conjugation is the clinically dominant approach for siRNA delivery to hepatocytes. Given the wide therapeutic index and excellent safety profile of these compounds, four chemically modified GalNAc-siRNAs have been FDA-approved (Givosiran [17], Lumasiran [16], Inclisiran [18], and Vutrisiran [66]) to treat liver-associated disorders.

## 3. Delivery Platforms to Enhance Oligonucleotide Therapeutics Intracellular Uptake

To reach their targets, oligonucleotides need to be delivered efficiently and in a productive manner inside the tissues/cells of interest. While PS-ASOs can spontaneously and productively enter cells without the need for a delivery system, they distribute mainly to the liver and kidneys after systemic administration, limiting their use to treat widespread diseases [67,68]. On the other hand, siRNAs are too large and charged to enter cells unassisted and require a delivery system to target any tissues after systemic injection. While both lipid nanoparticles and GalNAc conjugates are clinically validated and approved delivery strategies for liver targets, efficient delivery of oligonucleotides beyond the liver and kidneys is a fundamental obstacle preventing their clinical utility [14,15,16,17,18,19].

**Figure 4:**
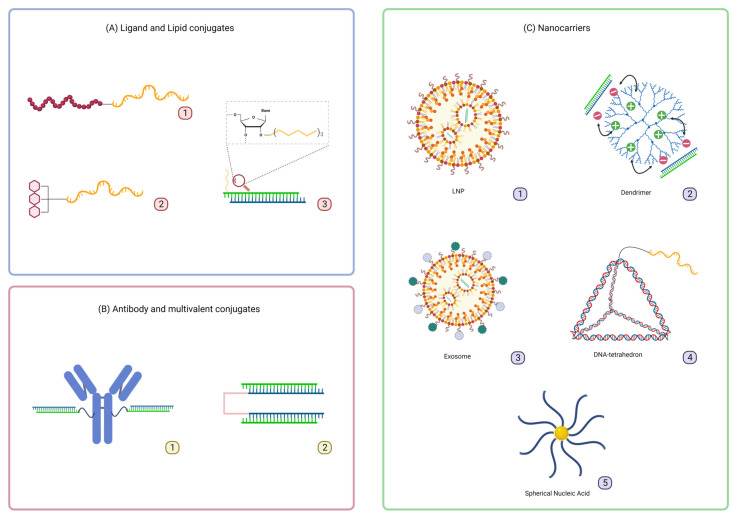
Schematic representation of mentioned delivery strategies applied to oligonucleotides. (**A**): (**A1**) Receptor agonist conjugates as exemplified with the GLP1R peptide agonist-ASO conjugate, (**A2**) GalNAc conjugate as found in liver targeting therapeutics, and (**A3**) lipid conjugates exemplified with the 2′-O-hexadecyl modified siRNA; (**B**): (**B1**) antibody–oligonucleotide conjugate, and (**B2**) divalent siRNAs scaffold; (**C**): (**C1**) LNPs as for instance used in patirisan, (**C2**) dendrimer–oligonucleotide complexes like PANAM, (**C3**) exosome delivery, (**C4**) DNA-nanoconstruct like the tetrahedron DNA origami, functionalized with an ASO, and (**C5**) spherical nucleic acid, made with a gold nanoparticle and packed ASOs. Figure created with BioRender.com, FU25OEFFUQ.

Oligonucleotide delivery systems have been thoroughly described previously [1,69], therefore, selected delivery approaches will be briefly mentioned in this review, including both direct ligand conjugation strategies and nanocarrier technologies (Figure 4).

Conjugation of a Glucagon-Like Peptide 1 Receptor (GLP1R) agonist to ASO enables efficient delivery to pancreatic beta cells, resulting in significant silencing of islet amyloid polypeptide in mice. The GLP1R conjugate shows not only cell selectivity, but also improves ASO productive uptake to intracellular compartments (Figure 4A1) [70,71].

Lipid conjugation has emerged as a delivery platform for oligonucleotides for systemic administration (Figure 4A3) [72,73]. It has been demonstrated that the chemical structure of the lipids significantly impacts oligonucleotide clearance, lipoprotein binding, tissue distribution, and efficacy [21,23,25,26,74]. Highly lipophilic lipids such as cholesterol tend to bind to low-density lipoprotein (LDL), driving oligonucleotide distribution to the liver. On the other hand, less lipophilic lipids such as docosahexaenoic acid (DHA) tend to bind to high-density lipoprotein (HDL) and accumulate in the kidneys. Even though the delivery to extra-hepatic tissues remains challenging, fatty acid conjugates including palmitic acid and docosanoic acid enable functional oligonucleotide delivery to the lungs, CNS, eyes, and muscles [22,24,75].

Antibody (Ab) conjugation is also applied to deliver both ASOs and siRNAs to extra-hepatic tissues (Figure 4B1) [76]. Conjugating an anti-transferrin receptor Ab (anti-TfR Ab) to PMO enables an increase in SMN2 mRNA level in the CNS when injected systematically to a spinal muscular atrophy mouse model. These data demonstrate that the cargo can cross the blood–brain barrier to deliver the drug efficiently [77]. In addition, multiple PMOs linked to an anti-TfR Ab induce an increase in dystrophin expression in skeletal muscle in *mdx* mice [78]. For siRNA delivery, the first example of antibody-mediated delivery was published in 2005 by the Liberman group against the HIV-1 capsid gene gag [79]. Since then, many leading pharmaceutical companies such as Avidity, Dyne, Tallac, Denali, and Genna Bio have been developing Ab-conjugated siRNAs for the treatments of various muscle disorders. Avidity’s AOC 1001, an anti-TfR conjugated siRNA, has entered clinical trials to treat DM1 disease, demonstrating the value of such an approach [80]. This compound shows >80% of mRNA silencing in muscles after a single-dose administration in mice [81]. Furthermore, siRNAs-Ab conjugates are being used in some instances as complexes with cationic peptidic sequences, conjugated to the Ab itself. An Ab targeting a prostate specific membrane antigen, a marker found in most prostate cancers, complexes siRNAs through a protamine sequence (a 30–65 amino acids long sequence, with >50% of arginine). This system shows a significative inhibition in the proliferation and the growth of cancerous cells in a castration-resistant prostate cancer mouse model [82].

Based on organic scaffolds, an interesting approach consists of linking multiple siRNAs or ASOs together, as a multimer vehicle to improve delivery (Figure 4B2) [83,84]. A Di-siRNA scaffold targeting SARS-Cov2 mRNA has been reported, showing interesting properties in terms of exposure and duration of the silencing effect in the lungs of mice, compared to the monovalent analogue [85].

Nanocarriers represent an important class of oligonucleotide delivery systems. These nanotechnologies can be of many forms, going from liposomes [86], peptides [87], dendrimers [88], exosomes [89], spherical nucleic acids (SNAs) [90,91,92], or DNA nanostructures [93], to name a few (Figure 4C). One advantage of this strategy is the possibility to control and fine-tune the biophysical properties of the carrier to enhance oligonucleotide cellular uptake, intracellular trafficking, and endosomal escape. The control of the particle size is particularly interesting when considering oligonucleotide pharmacokinetic properties and elimination through renal clearance [94]. In the case of SNAs, for example, the density of oligonucleotides conjugated to the gold nanoparticle can be controlled to impact the cellular uptake of the cargo [95]. Nanoparticles can either self-assemble as complexes, as in the case of LNPs and dendrimers, or be precisely synthesized as with DNA-based technologies. Exosomes are also interesting as vehicles for oligonucleotide delivery, they are found endogenously and shown to be more resistant and less toxic compared to LNPs. They were successfully engineered and used to deliver large materials like single-guide RNAs and even plasmids [96,97].

## 4. Overcoming Endosomal Barrier to Maximize Oligonucleotide Therapeutic Activity

Unlike small molecule therapeutics, oligonucleotide therapeutics are large polyanionic molecules, which do not readily diffuse across the cellular membrane. Both receptor and non-receptor targeted oligonucleotides are initially internalized by various endocytic mechanisms, depending upon cells and brought into the early endosomes (EEs) [27]. Oligonucleotides—whether non-conjugated, ligand-conjugated, or associated with nanocarriers—are gradually trafficked from the EEs to late endosomes (LEs) to downstream multivesicular bodies (MVBs) and finally to lysosome (LYs) for degradation (Figure 2) [98,99,100]. In this event, oligonucleotide therapeutics experience an environmental change in pH (7.4–4.5) as they travel along the endocytic pathway [31]. In addition, while oligonucleotides are stable in endosomal compartments, small amounts can be recycled back to the extracellular space through exocytosis [101]. Therefore, it is crucial for the oligonucleotides to escape from endosomes before lysosomal degradation and recycling events to maximize their therapeutic effect.

As oligonucleotides are polyanionic macromolecules, escaping the endosomal lipid bilayers to reach their intracellular targets represents a major obstacle, making the endosomal escape to be a rate limiting factor for the oligonucleotide therapeutic effect [31]. It has been reported that only 1–2% of GalNAc-PS ASOs and less than 0.3% of GalNAc-siRNA conjugates escape endosomes to engage with the target in vivo [28,102]. Therefore, there is an urgency to understand the mechanism of endosomal escape to enhance oligonucleotide release into the cytosol.

Only little success in attempts to improve the escape of oligonucleotides from endosomes has been reported. Although the use of endosomal escape domains (EEDs) represents a promising approach to enhance endosomal escape, their mechanism of permeabilizing endosomes may induce toxicity, narrowing the oligonucleotide therapeutic index [30,103,104,105,106,107]. Many of EEDs lead to endosomal bursting releasing a wide array of its luminal content into the cytosol, resulting in activation of the innate immune system and other toxic pathways [29]. Even though ~99% of oligonucleotide therapeutics remain entrapped in endosomes or lysosomes, recent evidence suggests that trapped oligonucleotides serve as a depot, enabling slow oligonucleotide leakage over the course of time and thus long duration of the effect [102]. Therefore, to solve the endosomal delivery problem, it is crucial to acknowledge the balance between enhancing endosomal escape for better efficacy, having a partial depot effect for long duration of response, and maintaining safety.

## 5. Strategies to Enhance Endosomal Escape

**Figure 5:**
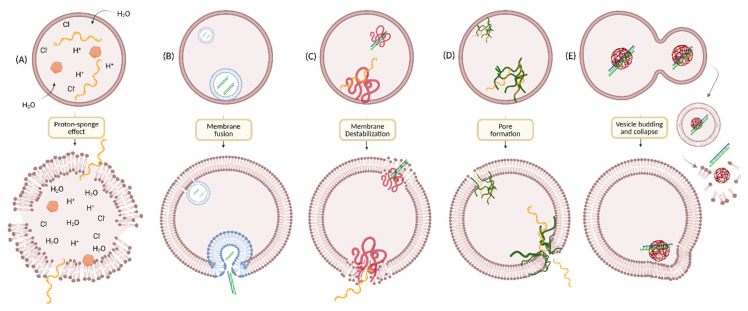
Proposed mechanisms of endosomal escape. (**A**) Proton-sponge effect: buffering polymers or small molecules induce increased inflow of protons, counterions, and water molecules into endosomes, resulting in high osmotic pressure and hence endosomal rupture; (**B**) membrane fusion: ionizable lipids containing fusogenic lipids fuse with endosomal bilayer and hence releases the oligonucleotides into cytosol; (**C**) membrane destabilization: polymers containing pH-sensitive scaffolds interact with anionic endosomal membrane and induce membrane destabilization; (**D**) pore formation: some endosomolytic peptides form pores in endosomal membrane, which allows escaping of oligonucleotides into cytosol. It can be of two types such as barrel-stave pore formation and toroidal pore formation. (**E**) Vesicle budding and collapse: some endosomolytic peptides induce budding and collapse of CPP-containing vesicles from endosomal membrane. Created with BioRender.com, JF25FK1RLE.

Microorganisms like viruses (enveloped or non-enveloped) and bacterial toxins are naturally built with efficient strategies to escape endo-lysosomal vesicles for their own benefit. Mostly, they escape endosomes via pore formation and/or membrane fusion [108,109,110]. However, it is unlikely to use viral carriers as oligonucleotide delivery systems as they are associated with cytotoxicity and potent immune responses on repeated dosing [111]. Understanding their mechanisms has inspired researchers to explore non-viral delivery systems to enhance endosomal escape efficiency. Even being a subject of intensive investigation for more than four decades, the mechanism by which oligonucleotide therapeutics escape the endosomes remain unknown, limiting the development of efficient delivery systems. Several hypotheses on how drugs escape endosomes have been reported and summarized in a review by D. Pei and M. Buyanova [112]. Briefly, endosomal escape can be triggered by either one or multiple simultaneous mechanistic events such as membrane fusion, pore formation, proton sponge effect, membrane destabilization, or vesicle budding and collapse, depending upon the chemical properties of different endosomal escape domains (Figure 5) [113,114,115,116,117,118,119,120,121,122].

The endosomal pH is associated with the stage of endosomal maturation. It decreases progressively from the cell surface (pH ~7.4) to pH 6.5 in early endosomes and then, pH 5.5 in late endosomes. This pH change in the endocytic pathway can facilitate the endosomal escape of oligonucleotides into cytosol by using pH-sensitive scaffolds, which can interact with the endosomal membrane and disrupt it at an acidic pH. Taking advantage of pH differences between the extracellular matrix and endosomal compartments, a variety of endosomolytic agents such as endosomal buffering polymers, endosomolytic peptides, small molecules, ionizable lipids, and cationic liposomes have been developed and used in gene delivery.

### 5.1. Endosomal Buffering Polymers

**Figure 6:**
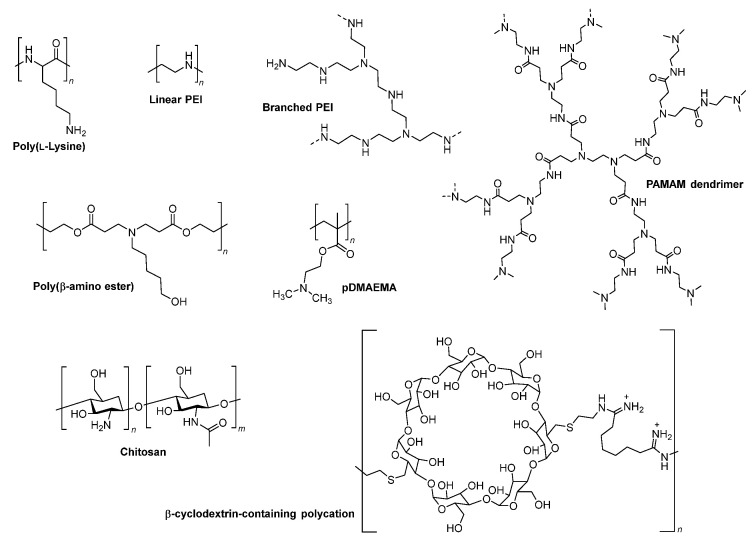
Chemical structures of selected endosomal buffering polymers that are commonly used in gene delivery studies. Poly(L-lysine) and polyethylenimine (PEI) are among the earliest used buffering polymers. Several other polymeric vectors, such as polyamidoamine (PAMAM) dendrimers, degradable poly(β-amino esters) (PBAEs), poly[(2-dimethylamino)ethyl methacrylate] (pDMAEMA), and various carbohydrate-based polymers (chitosan and β-cyclodextrin-containing polycations), have also been explored to improve safety and efficacy.

As the pH of endolysosomal compartments decreases progressively from the cell surface (pH ~7.4) to the LYs (pH 4.5), the cytosolic delivery of nucleic acids using pH-dependent cationic polymers or polyplexes has been of great interest [121,123,124]. Poly(L-lysine) (PLL) and polyethyleneimine (PEI) and other polymeric vectors (Figure 6) taken up via endocytosis can form non-covalent polyplexes or nanoparticles with oligonucleotides through electrostatic interactions [125,126,127]. These polyamine vectors undergo protonation at low pH in endosomes, which prevents further endosomal acidification by absorbing more protons and counter ions. It results in osmotic swelling, which eventually leads to rupturing of the endosomal membrane, allowing the oligonucleotide system to escape into cytosol (a process called the proton-sponge effect) (Figure 5A) [120,128]. The use of pH sensitive cationic polymers as transfectants and effective nucleic acid delivery systems in vitro has been widely explored. However, the requirement of a large excess of the polymers for effective transfection often induces substantial toxicity during in vivo applications. Chemical modifications of PLL and PEI have been investigated to enhance their nucleic acid delivery properties [129,130,131], for example, PEGylated PLLs and PEIs to improve tolerability, degradable disulfide crosslinked PEIs to reduce toxicity, and alkylated PEI to increase potency. In addition, several other polymers are evaluated to address toxicity and efficacy concerns, including poly(amidoamine) (PAMAM) dendrimers, poly(β-amino esters) (PBAEs), poly[(2-dimethylamino)ethyl methacrylate] (pDMAEMA), and various carbohydrate-based polymers (Figure 6) [132,133,134,135,136]. While substantial progress has been made in this area, designing safe and efficient pH-dependent cationic polymers that can enhance the endosomal escape of oligonucleotides is still needed.

### 5.2. Cell-Penetrating Peptides (CPPs)/Endosomolytic Peptides (EPs)

If evolution led eukaryotic cells to carefully manage the exchange of information between their external environment, the endosomal compartments, and their cytosol, viruses surely co-evolved to overcome these barriers [137,138]. A first example comes from HIV-1, which was found to easily cross the plasma membrane thanks to its trans-activating regulatory protein (TAT) [139,140]. It was discovered that only a small peptidic portion of TAT (Table 1), called the protein transduction domain (PTD) and rich in basic Arginine and Lysine amino-acids, could be synthesized and fuse to different cargos for efficient delivery inside cells [141,142]. Therefore, CPPs could be an interesting approach to overcome the endosomal barrier and increase drug delivery [143,144,145,146]. While getting inspiration from nature is elegant, the solution is not that simple.

In the case of cationic CPPs, their mode of action is still under debate. It was first thought that the peptides, like their parent proteins, would translocate directly to the cytosol through the bilayer cell membrane, in an energy-independent manner [147,148]. Further experiments show this is not really the case and that the peptides, just like oligonucleotides, are internalized through endocytosis. One hypothesis is that the positively charged peptide interacts with the anionic proteins found on the cell surface, such as the heparan sulfate proteoglycans, initiating the endocytic pathway in an energy-dependant manner (e.g., macropinocytosis) [149]. Once inside the endosome, cationic CPPs can possibly aid in endosomal escape via the proton-sponge effect or membrane destabilization [150], but according to the latest work, they seem to be more efficient at only penetrating the cell membrane [147]. While cationic CPPs are mainly used to deliver a neutrally charged backbone (i.e., PMO and PNA), a siRNA conjugated to TAT, penetratin, or LAH4-L1 peptides shows effective gene silencing in vivo and in vitro, respectively (Table 1) [151,152]. The LAH4-L1/siRNA complex enters the cell through a cholesterol-dependent process and may escape from endosomes via multiple pathways [153].

Amphiphilic peptides, as illustrated by the HA2 protein from influenza viruses or the synthetic GALA peptide designed by Szoka group (Table 2), have a different mode of action for initiating endosomal release [154,155]. Their sequence is composed of glutamic acid amino-acids that, when the pH of the compartment drops along the endosomal pathway, adopts a helical conformation [156]. This change of conformation disrupts the endosomal membrane, leading to the leakage and release of the cargo. The capacity of amphiphilic EPs to induce endosomal escape seems hampered when they are directly and covalently conjugated to the cargo [147,157]. The Harashima group discovered that liposomes modified with GALA peptides improve siRNA transport into cells and that combining GALA peptides with PPD (PEG-peptide-DOPE) in lipid-based nanoparticles improves endosomal escape, siRNA delivery, and gene silencing [158]. In addition, various other amphiphilic EPs, such as INF7, TAT-HA2, bPrPp, and aMel, have been developed to increase endosomal escape in gene delivery systems [143]. Co-injection of a GalNAc-conjugated siRNA and a GalNAc-INF7 peptide shows a significant increase in mRNA gene silencing compared to administration of GalNAc-siRNA alone, proving the efficacy of such a strategy [102].

“Penetration Accelerating Sequence” (PAS are short hydrophobic peptides mainly composed of tryptophane and phenylalanine (Table 1). It was first discovered that the attachment of a hydrophobic tail (-GKPILFF-derived from the cathD protein) to an arginine-rich sequence was accelerating and increasing the cytosolic translocation of fluorescent cargos [163]. When accumulating inside the endosomal compartments, the hydrophobic moieties insert in the lipid bilayer and locally destabilize the membrane, releasing their content inside the cytosol [165]. Studies show that a combination of the TAT domain with a hydrophobic tail of amino acid increases the cargo delivery in cytosol both in vitro and in vivo [164,166].

### 5.3. Small Molecules

Compared to the important size and the polyanionic character of oligonucleotides, most small molecules have the advantage of diffusing freely across the cellular membranes and subcellular compartments. Several groups have performed a library screening to seek compounds able to interact with the endosomal pathway and influence the escaping of oligonucleotides [167,168]. This family of molecules has different denominations: endosomolytic compounds, endosomal escape enhancers, and oligonucleotide enhancing molecules (OECs). In this review, they will be named OECs.

**Figure 7:**
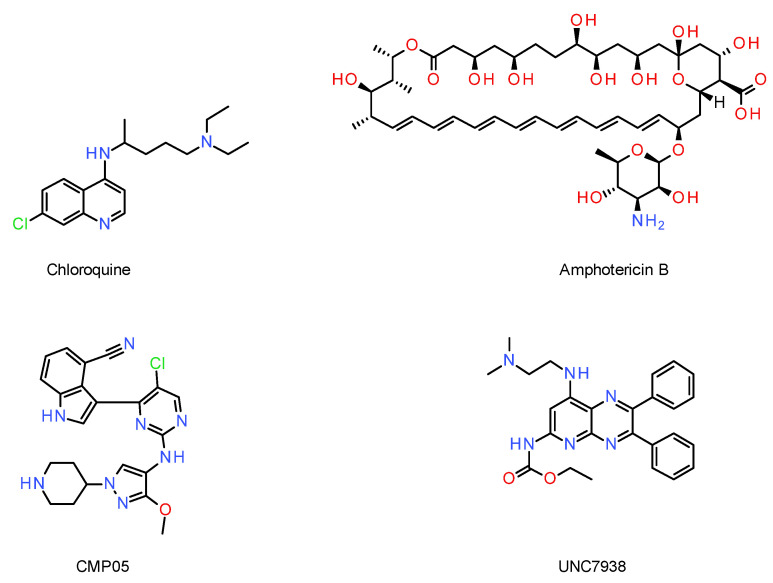
Few examples of chemical structures of OECs used in oligonucleotide delivery.

One of the most well-known examples is the chloroquine (Figure 7) that, once in acidic vesicles, becomes protonated and generates a “proton-sponge effect”, swelling the compartment and allowing leakage of the endosome compartment in the cytosol [169]. Operating the same way, CMP05, a compound characterized by Bost et al., shows a higher endosomal rupture effect than the chloroquine and at lower doses (Figure 7). CMP05 was successfully used to improve SSO delivery and could be a potential adjuvant for oligonucleotides [168].

OECs can also induce permeation at different stages of the endosomal pathway, allowing the release of oligonucleotides in the cytosol [167]. This is for example the case for UNC7938 (Figure 7), a molecule found by Juliano et al. that acts between the early endosome and lysosomal stages. It is hypothesized that the improved endosomal release happens in the LEs/MVBs, where oligonucleotides have a higher chance of escaping [170].

OECs interacting physically with the endosomal membrane and inducing membrane permeability by forming transmembrane pores have been studied. The antifungal drug amphotericin B is one example and was successfully used to deliver siRNAs and ASOs into the cytosol (Figure 7) [171].

While OECs show promise to enhance oligonucleotide endosomal escape, they bear important challenges. (i) They have their own pharmacological actions and their own side-effects, resulting in potential toxicity. (ii) The non-specific swelling of vesicles induced by OECs might release undesired products (degraded proteins and exogenous material), leading to additional toxicity. (iii) The timing of dosage is important, should the OECs be co-injected with the drug or delayed by an X amount of time? It becomes challenging as the OECs and the oligonucleotides have distinct pharmacokinetic/pharmacodynamic properties in vivo. (iv) Different tissues/cell lines react differently to OECs, and thus a careful optimization is required to reach an efficient OEC–oligonucleotide cocktail [169].

### 5.4. Cationic Liposomes

As previously mentioned, LNPs offer great opportunities to efficiently deliver oligonucleotides. This technology has already been used in three nucleic acid FDA-approved drugs: Patisiran, an siRNA drug that treats hereditary disease transthyretin-mediated amyloidosis, and two SARS-CoV2 mRNA vaccines, Tozinameran and Elasomeran [172,173,174].

**Figure 8:**
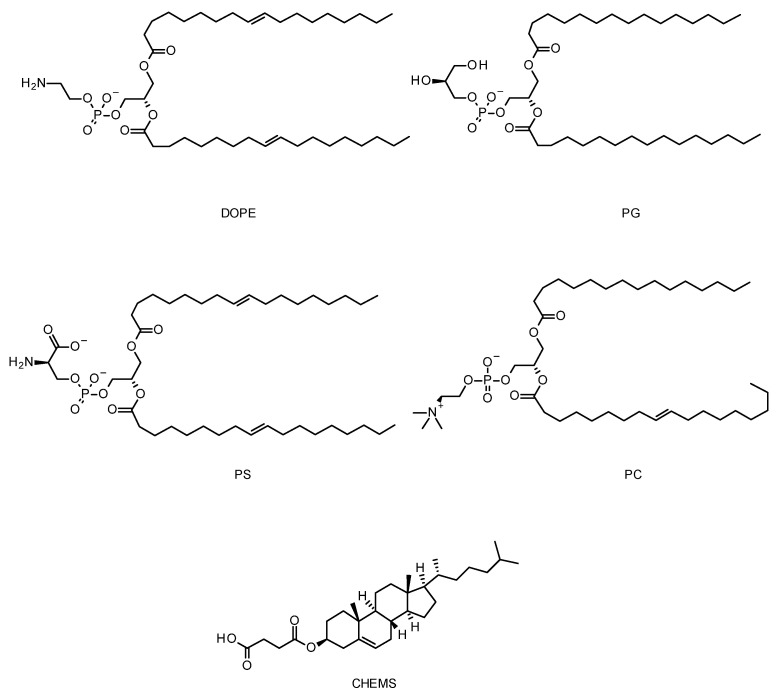
Chemical structures of some examples of lipids used in LNPs. Dioleylphosphatidylamine (DOPE), phosphatidylglycerol (PG), phosphatidylserine (PS), phosphatidylcholine (PC), and cholesteryl hemisuccinate (CHEMS).

The lipid composition of the particles can be carefully selected and designed to improve parameters including drug encapsulation, particle size and stability, but also to confer extra properties such as endosomal escape enhancement [175,176,177]. In that regard, cationic lipids are particularly interesting as vesicle components. pH-sensitive cationic liposomes get destabilized at acidic endosomal pH, leading to their escape from endosomes through destabilization or their fusion with the endosomal membrane. Dioleylphosphatidylamine (DOPE) is one the key components of pH-sensitive liposomes, in addition to weakly acidic amphiphiles such as cholesteryl hemisuccinate (CHEMS), phosphatidylglycerol (PG), phosphatidylserine (PS), and phosphatidylcholine (PC) (Figure 8) [144,178,179,180]. In an acidic environment, DOPE undergoes phase transition from a lamellar phase to an inverted hexagonal phase, which leads to the destabilization of the endosomal membrane [181]. Alternatively, an acid-cleavable group can be introduced to increase the transfection efficiency of the liposomes. With this aim, for example, the primary amine of DOPE has been modified with citraconic anhydride to form citraconyl-DOPE. The citraconyl group cleaves at a low pH to reform DOPE to its initial form in the endosomes [182]. Based on their pH-sensitive behaviour, various liposomes, such as GALA-modified liposomes and PEGylated YSK05-MEND, have been used for effective delivery of siRNA to the target cell [158,183].

## 6. Importance of Linker Chemistry in Drug Delivery

As mentioned previously, chemical modifications are required to improve oligonucleotide drug-like properties but are not sufficient to efficiently deliver oligonucleotides to the targeted cells/tissues [184,185]. While a variety of deliver systems has been explored, direct conjugation of oligonucleotides to ligands—such as lipids [21,25,186], peptides [70,187,188,189], aptamers [190], antibodies [191], and sugars (e.g., GalNAc) [192,193]—is emerging as the clinically dominant delivery strategy. Therefore, combining chemical modifications and conjugation approaches gives opportunities to maximize oligonucleotide delivery to the tissues of interest. GalNAc conjugated to fully chemically modified siRNA has revolutionized the development of oligonucleotide therapeutics to treat liver diseases [194]. Furthermore, several groups have pioneered the use of peptide–PMO (PPMO) conjugates for the treatment of various diseases, most notably for dystrophin splice-switching in regard to DMD and SMA [189,195].

**Figure 9:**
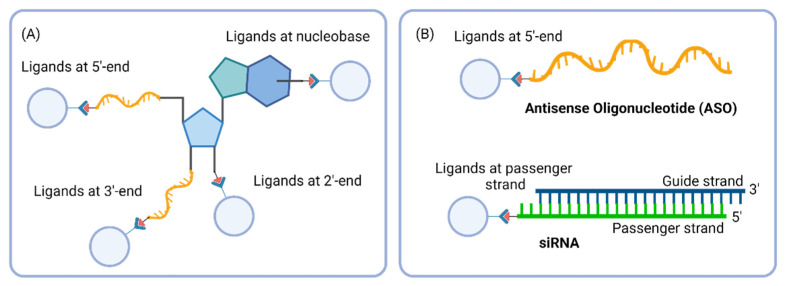
(**A**) Ligand conjugation sites in oligonucleotides. (**B**) Preferred ligand site conjugation in ASO and siRNA. Created with BioRender.com, LN25GKFR3K.

In conjugated oligonucleotides, ligands can be attached covalently via different linkers at various positions, generating a wide range of well-defined conjugated compounds with distinct properties. Ligand conjugation can generally occur at the 3′ and 5′ ends on the hydroxyl terminal groups, or at any nucleotides on the 2′-hydoxyl groups and nucleobases (Figure 9A). It has been preferred to link conjugates at the termini of oligonucleotides to minimize base-pairing interference. In siRNA, the passenger strand is generally conjugated to the ligand, because, although the duplex is loaded into RISC complex, only the guide strand is functional for mRNA hybridisation and degradation. Therefore, ligand-conjugated passenger siRNA are expected to have no or little influence on RNAi activity (Figure 9B).

The employed linkers to conjugate the ligands to the oligonucleotides dictate the stability of ligand–oligonucleotide conjugates in plasma and play a significant role in their biophysical properties, pharmacokinetic behaviour, and therapeutic efficiency. A well-designed linker should be stable in plasma to avoid premature conjugate cleavage and enable efficient release of active drug at the targeted site of action. Both length and nature of the linkers can affect the therapeutic property of oligonucleotides. Therefore, optimizing the structure of the linker is essential to maximize the conjugated-oligonucleotide activity. Schematically, linkers are divided into two categories: non-cleavable and cleavable linkers.

Non-cleavable linkers are generally more stable and the conjugates with non-cleavable linkers remain intact for a prolonged time. The use of these linkers could avoid the premature release of a drug in circulation and could be beneficial in conjugated compounds where it plays a role in drug activity [196,197]. It could also be useful for drugs that are required to bind two different targets at the same time in order to bring them in close proximity to achieve the desired pharmacological effect [198]. Unlike cleavable linkers, non-cleavable linkers do not have a particular weak bond in their structure that could be cleaved by enzymes, photo-irradiation, or change in pH. Therefore, the linker should be attached to an appropriate position to ensure no significant interference with the binding of the drugs to their targets. In addition, the hydrophobicity and length of the linker should be optimized to ensure optimal water solubility and distance between ligand and drug molecule to maximize drug activity.

Cleavable linkers can be described as chemical entities with two functional heads joined together through a cleavable bond, which can be cleaved in a controlled fashion to release at least two different molecular species. In the early 1990s, the development of various cleavable linkers triggered the rapid expansion of solid-phase organic synthesis, resulting in the smooth release of the payload from its solid support [199]. Cleavable linkers are broadly classified into enzymatic, physicochemical, or chemically labile linkers depending on the conditions used for their cleavage [200]. This review mainly focuses on the chemically cleavable linkers used in drug delivery. Chemically cleavable linkers such as acid-cleavable and reducible linkers have been thoroughly explored and are undoubtedly a part of a general synthetic chemist toolbox. As the harsh conditions required by organic synthesis linkers are unsuitable for biochemical processes, chemists started to reassess potential functional groups and cleavage reactions that could satisfy various challenging constraints. Linkers should be labile in mild conditions, resistant to labelling and purification of conjugates, easy to synthesize and high yielding at low concentrations, and require the use of bio-orthogonal reagents.

## 7. Cleavable and Non-Cleavable Linkers in Oligonucleotide Conjugates

Linkers of various length, polarity, stability, and flexibility are used for different types of oligonucleotide conjugates and the choice of linker structure is mostly driven by the desired mode of action. A non-cleavable linker is preferred over a cleavable linker when the stability of the conjugate is more important than the fast release of the drug. However, stable linkages may hinder the therapeutic activity of oligonucleotides. Once internalized, bulky ligands can interfere with the loading of oligonucleotides in biological machinery, such as RISC or RNase H, or can directly impact target binding.

Cleavable linkers, depending on their nature, offer a greater degree of control over loaded oligonucleotides’ release mechanism. While it is unclear if ligand conjugation impacts oligonucleotide endosomal escape, dissembling ligands from oligonucleotides could be beneficial, where free oligonucleotides can be more therapeutically active. Therefore, cleavable linkers have gained immense importance and become a subject of investigation in the field of drug discovery [200,201].

**Figure 10:**
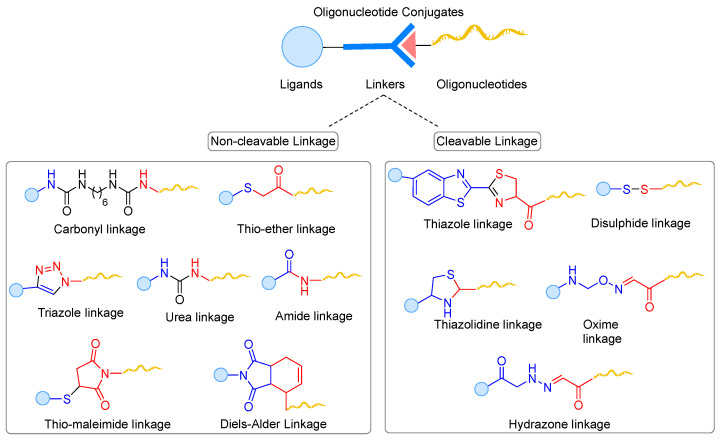
Commonly used linkages for ligand-oligonucleotide conjugation. Note: Generalized linker structures are shown in this figure, which may be different from original linker structures.

Over the years, different types of ligation methods for tethering the oligonucleotides have been explored (Figure 10) [202,203]. The most used non-cleavable (i.e., amide, triazole, and maleimide linkages) and chemically cleavable (i.e., disulfide and pH-sensitive linkages) linkers in oligonucleotide therapeutics are described in more details in this section.

### 7.1. Non-Cleavable Linkages

#### 7.1.1. Amide Linkages

Generally, the amide linkages are formed by a chemical reaction between an amino group present on the linker attached to the oligonucleotide and an activated carboxylic acid group bearing by the ligand. It is very common to use amide linkages in the design of oligonucleotide conjugates as they are biocompatible and have minimal influence on the physiochemical properties of oligonucleotide conjugates. However, using amide linkages imposes certain limitations. (i) The nucleophilicity of amino groups is pH dependent, which results in low reactivity towards electrophile at a lower pH; and (ii) the conjugation reaction in solution phase involves the use of carbodiimide-activated intermediates, which can lead to side reactions and low coupling yields due to the instability of activated esters in aqueous media. Therefore, a careful optimization of conjugation conditions is required to efficiently synthesize conjugated oligonucleotides using amide linkage.

**Figure 11:**
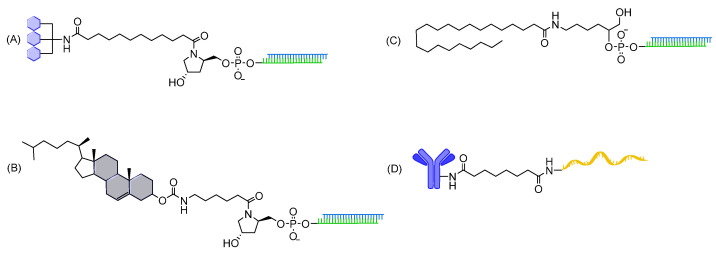
Oligonucleotide conjugates containing amide linkages. (**A**) GalNAc-siRNA conjugate; (**B**) cholesterol-siRNA conjugate; (**C**) fatty acid-siRNA conjugate; and (**D**) antibody-ssDNA conjugate.

The GalNAc conjugate has been successfully linked to siRNAs via amide linkage (Figure 11). These compounds demonstrate robust and prolonged silencing in hepatocytes and have been FDA-approved to treat liver diseases [9]. In addition, several studies report in vivo therapeutic activity in multiple extra-hepatic tissues when using lipid (e.g., cholesterol and fatty acids)-conjugated siRNAs containing amide linkages [22,23,25,204,205]. Recently, a robust ‘plug and play’ antibody conjugation of succinimidyl-functionalized oligonucleotides containing stable amide linkages enables to develop highly sensitive bioassays for specific epitopes in laboratory and clinic [206].

#### 7.1.2. Triazole Linkages

**Figure 12:**
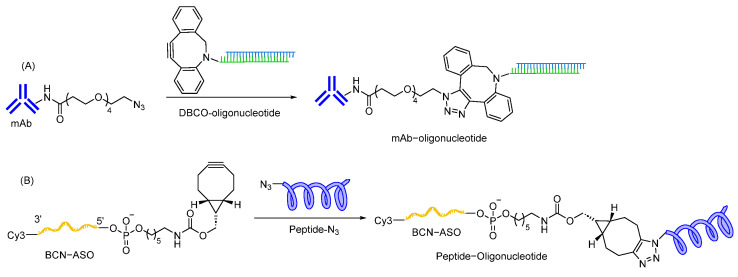
Oligonucleotides containing triazole linkages formed using SPAAC reaction. (**A**) mAb-siRNA conjugates containing DBCO as strained alkyne; (**B**) Ang II peptide-ASO conjugate containing BCN as strained alkyne.

Click chemistry is an attractive approach for the synthesis of oligonucleotide conjugates due to its high stability, high specificity, efficiency, and fast reaction time. A click reaction between azide and alkyne can be easily carried out with or without a copper catalyst in solution phase [207,208]. Copper-based catalysts are not preferred as it is difficult to remove copper residue after the reaction, resulting in cytotoxicity. Therefore, copper-free click chemistry such as strain-promoted alkyne-azide coupling (SPAAC) using cyclooctyne-based scaffolds is a more suitable approach for oligonucleotide conjugation. Chemical entities to introduce alkyne/cyclooctyne groups during oligonucleotide synthesis are commercially available, facilitating ligand conjugation [209,210]. SPAAC using dibenzocyclooctyne (DBCO) as a strained alkyne has been applied in several antibody–oligonucleotide conjugates utilized for the preparation of targeted anticancer agents [211,212] (Figure 12), multiplex protein detection assays [213,214,215], and epigenomic profiling [216]. Recently, Ang II peptide–ASO conjugates have been successfully synthesized via SPAAC using bicyclo [6.1.0] nonyne (BCN) as a strained alkyne for the targeted delivery of ASOs through an Angiotensin Type 1 Receptor [217].

#### 7.1.3. Maleimide Linkages

**Figure 13:**
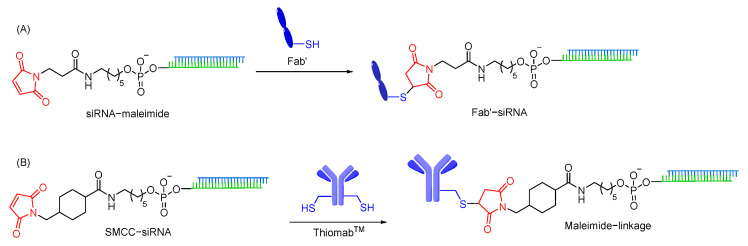
Oligonucleotide conjugates containing maleimide linkages. (**A**) Reaction of sulfhydryl-containing anti-CD71 Fab fragment with maleimide modified siRNA. (**B**) Conjugation of Thiomab^TM^ antibodies with siRNA using non-cleavable SMCC linker.

Thiol-maleimide linkages are widely used to conjugate oligonucleotides when non-cleavable linkers are required for oligonucleotide delivery. A maleimide moiety can be easily introduced into the ligand or oligonucleotide using succinyl spacers [218]. The maleimide motif acts as a Michael acceptor and rapidly reacts with the free thiol group to form thiol-maleimide linkage. As this linkage can be prepared in an aqueous buffer without a catalyst, thiol-maleimide linkage has become a commonly used linkage to conjugate oligonucleotides with various ligands, such as antibodies [219], and cell penetrating peptides [203,220,221]. However, thiol-maleimide linkages are susceptible to in vivo reduction, resulting in premature release of oligonucleotides from the ligand [222,223]. The use of retro-Michael ring opening of thiol-maleimide linkage is an alternative approach to overcome the potential reduction possibilities [203,224,225]. Anti-CD71 Fab antibody–siRNA conjugates (ARCs) using thiol-maleimide linkage enable to achieve significant gene silencing in muscle tissues [226]. Furthermore, siRNA is conjugated with Thiomab^TM^ antibodies, containing engineered site-specific cysteine, using SMCC linker that contains thiol-maleimide linkage. It is reported to induce gene silencing in various cell lines (Figure 13) [227].

### 7.2. Chemically Cleavable Linkages

#### 7.2.1. Disulfide Linkages

The disulfide linkages are generated by oxidative coupling of two free thiol groups, usually by pre-activation of one of the reacting thiols to avoid formation of homodimeric byproducts. While the disulfide linkages are sufficiently stable for storage and handling, they are instable under a reducing environment in endosomal compartments [228]. Hence, disulfide linkages can be employed as a cleavable linker for selective ligand-oligonucleotide decoupling into the cells, liberating free oligonucleotides, which might be more preferable to prevent target interference caused by the ligands. Usually, thiol linkers are commercially available for attachment at the 5′-end of oligonucleotides during solid-phase synthesis. However, it can also be attached to commonly used amino linkers in solution phase, such as use of succinimidyl 3-(2-pyridyldithio)propionate, a thiol-amine hetero-bifunctional crosslinker [229]. In addition, versatile phosphoramidite linkers containing disulfide linkage and terminal tosyl handle can be used to conjugate oligonucleotides [230].

**Figure 14:**
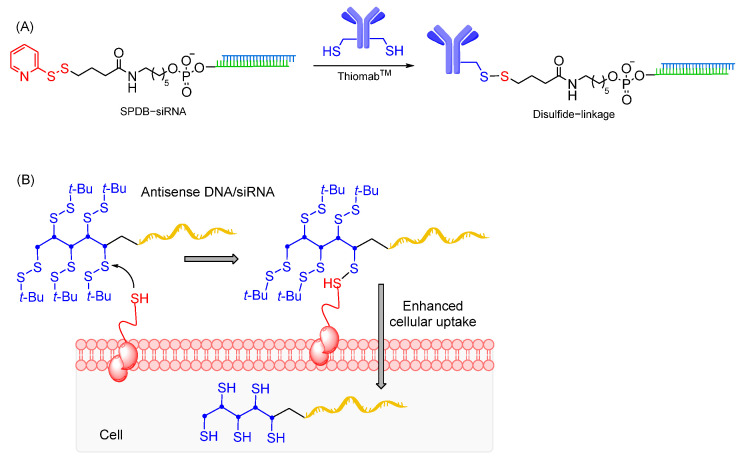
(**A**) Conjugation of Thiomab^TM^ antibodies with siRNA using cleavable SPDB linker; (**B**) conjugation of oligonucleotides with disulfide units enhances cellular permeability through disulfide exchange reactions with the thiol group on the cellular surface.

Disulfide linkages are commonly utilized in peptide–oligonucleotide conjugates having a cysteine amino acid as thiol handles to achieve efficient coupling [231,232]. Also, conjugation of siRNAs with Thiomab^TM^ antibodies, containing engineered site-specific cysteine, have been successfully carried out using an SPDB cleavable disulfide linkage (Figure 14A). These compounds support strong gene silencing, demonstrating that SPDB linkers do not impact conjugate properties [227]. While disulfide linkages allow ligand conjugation, attachment of disulfide entities to oligonucleotides without ligand could also be a strategy to enhance oligonucleotide cellular uptake. Abe and co-workers have recently reported that disulfide units conjugated at the oligonucleotide terminus enable agile cytosolic internalization of antisense DNA and siRNA, possibly through disulfide exchange reactions with the thiol groups on the cellular surface (Figure 14). Gene silencing effects of disulfide oligonucleotides were found to be comparable with those of lipofection-mediated oligonucleotides in vitro, notably with no cytotoxicity [233]. In addition, disulfide modified ASOs shows efficient exon skipping in *mdx* myotubes through enhanced membrane permeability and nucleus internalization [234].

Although disulfide linkage is reported to be an effective approach to synthesize oligonucleotide conjugates, its preparation offers several drawbacks, namely, (i) use of expensive protected thiol and disulfide functionalities on both coupling partners; (ii) site-selective conjugation in the presence of multiple reactive thiol groups; and (iii) necessity of removing acid labile protecting groups [235,236,237]. Moreover, regardless of oxidative environment, the presence of cysteine and glutathione in systemic circulation may cleave disulfide linkages to a certain extent after a prolonged circulation time, resulting in a potential decrease in oligonucleotide delivery.

#### 7.2.2. pH-Sensitive Linkages

**Figure 15:**
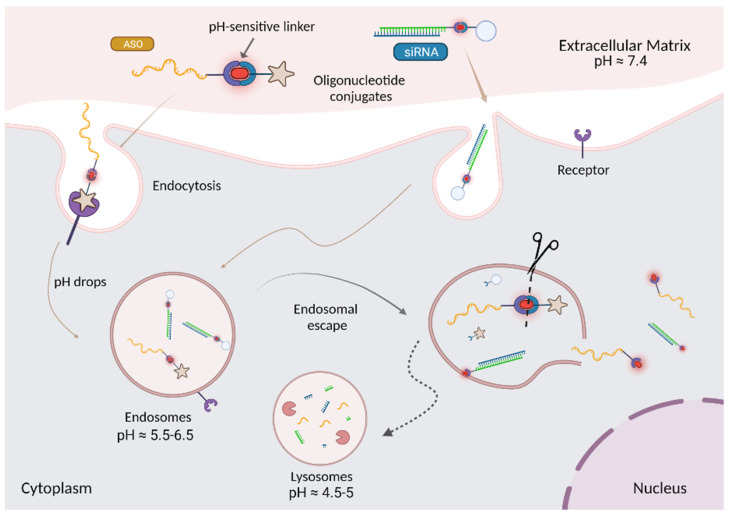
Trafficking of oligonucleotide conjugates containing pH-sensitive linker via endocytosis. The conjugates are internalized into the endosomal pathway by gymnotic uptake or by receptor-mediated uptake. pH-sensitive linkers are relatively stable in extracellular matrix at pH 7.4 and labile in endosomal compartments at pH 5.5–6.5. They can selectively release the oligonucleotide in endosomes and hence, can potentially enhance the endosomal escape of oligonucleotides. Created with BioRender.com, JQ25FK563G.

The pH-sensitive linkers are a type of chemically cleavable linkers, that are designed to selectively cleave under acidic environment, such as in endolysosomal compartments or tumour microenvironment [238]. At physiological pH 7.4, these linkers are relatively stable and hydrolyse very slowly, while the rate of hydrolysis is faster under acidic environment. Therefore, taking advantage of the pH difference between plasma and endosomal compartments/tumor site, they can selectively release the drug into the targeted site or in endosomes to potentially increase their intracellular escape (Figure 15) [239].

**Figure 16:**
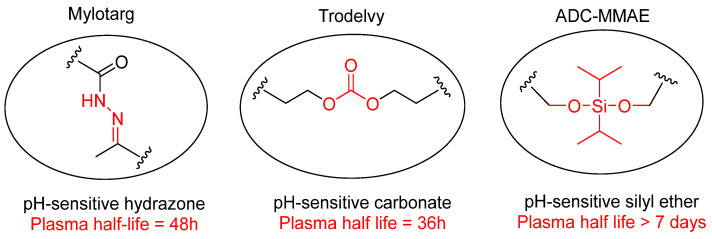
General chemical structure of examples of pH-sensitive linkers used in ADCs.

The use of pH-sensitive linkers is well known in Antibody–Drug Conjugates (ADCs) [201]. Pfizer developed the first FDA approved ADC, gemtuzumab ozogamicin (Mylotarg), which employed a pH-sensitive *N*-acyl-hydrazone linker. It was voluntarily withdrawn in 2010 mainly for causing liver toxicity due to the presence of an unstable hydrazone linker, however, it still has dose-limiting hepatoxicity and was approved again in 2017 after redesign [240,241,242,243]. The redesigned phenylketone-derived hydrazone linker was hydrolysed with a half-life, *t*_1/2_ = 48 h, in human and mouse plasma, demonstrating the importance of well engineering the linker chemistry to develop efficient drugs (Figure 16). Later, Gilead sciences used another carbonate-based pH-sensitive linker to conjugate cytotoxin SN-38 with humanized anti-Trop2 monoclonal antibody (mAb) (Figure 16). The resulting Sacituzumab govitecan (Trodelvy) gained Breakthrough Therapy designation from the U.S. FDA and approval in 2020 for adult patients with metastatic triple-negative breast cancer (TNBC) [244]. However, the serum stability of pH-sensitive carbonate linker was also found to be less than expected with *t*_1/2_ = 36 h and again left a question mark on its mechanism of action [244,245]. Therefore, the structural category of pH-sensitive linkers is still limited and their stability in plasma has been a major concern.

In search of more stable pH-sensitive linkers, the Zhou group developed a novel silyl ether-based pH-sensitive linker to synthesize an ADC carrying cytotoxic monomethyl auristatin E (MMAE, Figure 16) with *t*_1/2_ > 7 days in human plasma [246]. This design improves the stability of pH-sensitive linkers as well as possesses strong cell inhibitory activity in HER2^+^ cell lines with IC_50_ = 0.028–0.170 nmol/L. In addition to the improved stability, the Zhou group claimed effective release of the drug at the target site, appropriate efficacy, and controlled therapeutic toxicity.

Although ADCs can achieve site selectivity through antibody and systemic stability through a linker, non-specific toxicity can still be observed due to non-selective conjugated small molecules. This brings into light antibody–oligonucleotide conjugates (AOCs), which combines the appropriate precision of ASOs and siRNAs with the targeted delivery of antibodies, thus synergizing the advantages of both technologies. Dyne Therapeutics uses a cleavable linker for their pipelined AOC conjugates to release oligonucleotides in the endolysosomes [76].

**Figure 17:**
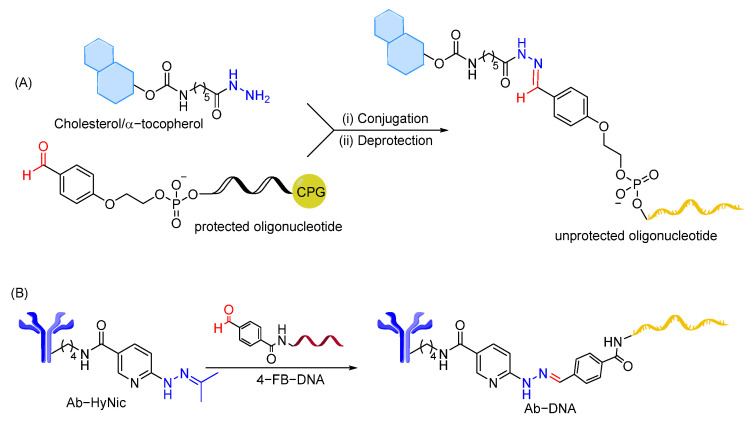
Oligonucleotide conjugates containing pH–sensitive hydrazone linkages. (**A**) Solid–phase synthesis of 5′–liphophilic conjugates of oligonucleotides via hydrazone bond formation. (**B**) Hydralink techniques for antibody–oligonucleotide conjugates preparation.

As in ADCs, hydrazone linkage has been successfully used to conjugate cholesterol and α-tocopherol lipids to siRNA targeting MDR1 mRNA, mitochondrial antireplicative RNAs targeting control region of mouse mtDNA, and guide RNAs for Mito-CRISPR system (Figure 17A) [247]. The lability of the hydrazone bond at different pHs was tested using siRNA conjugates, showing stability of the linkage at neutral pH and high hydrolysis rate at endosomal pH. In addition, antibody–oligonucleotide conjugates containing hydrazone linkage are used for sensitive assays to determine protein concentrations, where hydrazone bonds are reported to be stable enough at physiological pH (Figure 17B) [248]. An attempt to employ hydrazone linkage to conjugate a glyoxylyl-modified DNA oligonucleotide with N-terminal hydrazide peptides showed instability of glyoxylyl and hydrazide groups during synthesis and purification, demonstrating the limitation of hydrazone linkage in peptide–oligonucleotide conjugation [203,249].

**Figure 18:**
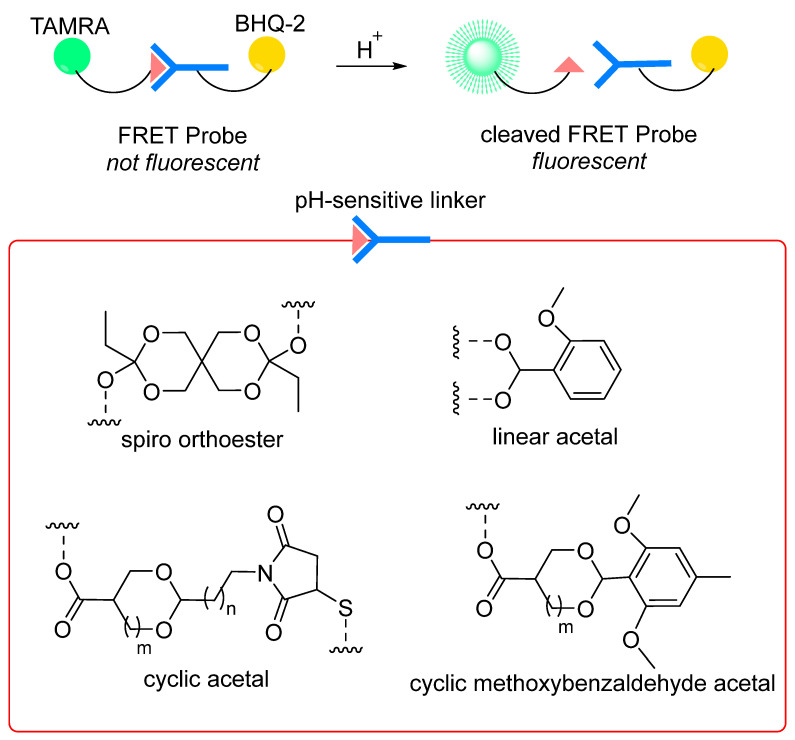
Design of FRET probes incorporating acetal-based pH-sensitive linkers.

The use of pH-sensitive linkers in the oligonucleotide therapeutics field remains limited and designing new linkers is required to effectively release oligonucleotides in endosomes/targeted sites. Recent developments in acid-sensitive prodrugs focus on increasing the difference in hydrolytic cleavage rate of linkers between physiological and endosomal/target site pH values, with the only aim to enhance the selectivity of hydrolysis. In light of this strategy, the Wagner group measured the hydrolysis rate in aqueous buffer solution at pH 7.4 (to stimulate plasma conditions) and 5.5 (to stimulate endosomal pH) for various functional groups in dummy compounds, such as spiro di-orthoester, dialkyl acetal, ketal, β-thiopropionate, oxazilidine, imidazolidine, acetal, hydrazone, acylhydrazone, 1,3-dioxolane, siloxane, oxime, carbamates, etc. Using a FRET-based experiment (Figure 18), the different bonds were classified based on their selective cleavage at acidic pH compared to neutral pH (*t*_1/2_ at pH 5.5 vs. *t*_1/2_ at 7.4) [250]. It has been claimed that hydrolysis of pH-sensitive linkers can be considered highly selective if the ratio *t*_1/2_ (pH 7.4):*t*_1/2_ (pH 5.5) is more than 15 [251]. The ratio *t*_1/2_ (pH 7.4):*t*_1/2_ (pH 5.5) could then be used as one of the criteria to evaluate selectivity of pH-sensitive linkers. Among the evaluated pH-sensitive linkages, the spiro-orthoester linker (SpiDo) was reported to exhibit 23-fold faster hydrolysis at pH 5.5 than at physiological pH and show more selective endosomal release in different cell lines [251]. Building on the results from the SpiDo linker and linear acetal linker, cyclic acetal systems have been developed with the idea of developing pH-sensitive amine-to-thiol cross linkers. However, these linkers show high stability under acidic conditions (even at pH < 1), limiting their use as pH-sensitive linkers [225,252]. Recently, a novel class of cyclic methoxybenzaldehyde acetals demonstrates high stability in neutral pH/plasma and cleavage at acidic pH [253]. When installed as a pH-sensitive linker connecting the MMAF drug to the antibody trastuzumab, significant potency in vitro and in vivo was observed, indicating that cyclic methoxybenzaldehyde acetals are excellent candidates to be used as pH-sensitive linkers for drug release applications where endocytosis is expected.

**Figure 19:**
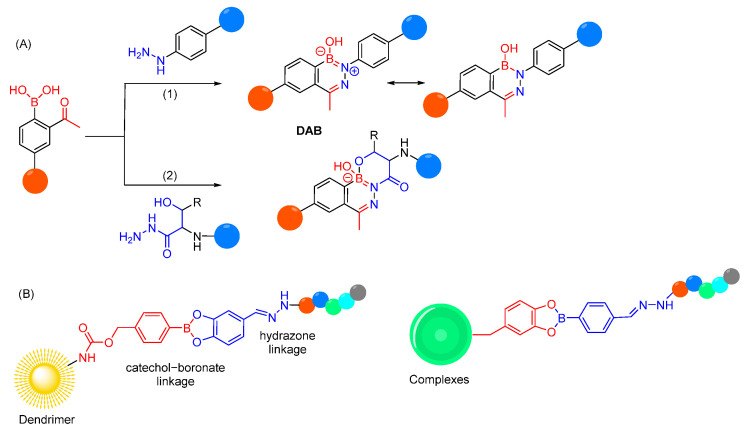
(**A**) Improvement in hydrolytic stability of aryl hydrazones by introducing a boronic group in the ortho-position. (**B**) Dual-responsive bioconjugates, containing two dynamic covalent linkages (hydrazone and catechol–boronate), for cytosolic delivery of impermeable peptides.

Conceivably, selectivity and reactivity of a linker can be influenced by the introduction of specific functional groups, which can help the linker chemistry in either of two ways, i.e., by improving the stability of hydrolytic labile bond in physiological pH, or by improving the lability of hydrolytic labile bond in acidic pH [254]. For example, based on high selectivity in hydrolysis of iminoboronates in physiological pH and low pH values, it is reported that introducing a boronic group in the ortho-position of aryl hydrazones can increase their hydrolytic stability in plasma by forming a dative N-B bond (Figure 19A) [255,256,257,258]. The reaction of *O*-acetyl phenolboronic acid with arylhydrazide does not produce the anticipated acylhydrazone but yields a six-membered benzodiazaborine ring (DAB). The latter shows remarkable serum stability and hydrophilicity due to its zwitterionic nature [259]. Further stabilization of the diazaboronate bond by an additional B-O bond was recently observed through the generation of *N*,*O*-bidentate ligands (Figure 19A) [260]. So far, DABs are mostly used for fast assembly of highly stable bioconjugates, but could offer opportunities to stabilize pH-sensitive cleavable linkers in plasma without impacting its cleavage in endosomes or could be used as a stable version of traditional *N*-hydrazone linkers [261].

pH-sensitive hydrazone linkages have also been employed in polymeric complexes and dendrimers in addition to the catechol–boronate linkages, which are also reversible and responsive to pH and redox environment. The dual-responsive bioconjugates containing these two dynamic covalent linkages are used for cytosolic delivery of impermeable peptides, with a controlled release of peptides under endosomal acidity or reactive oxygen species (Figure 19B) [262,263].

**Figure 20:**
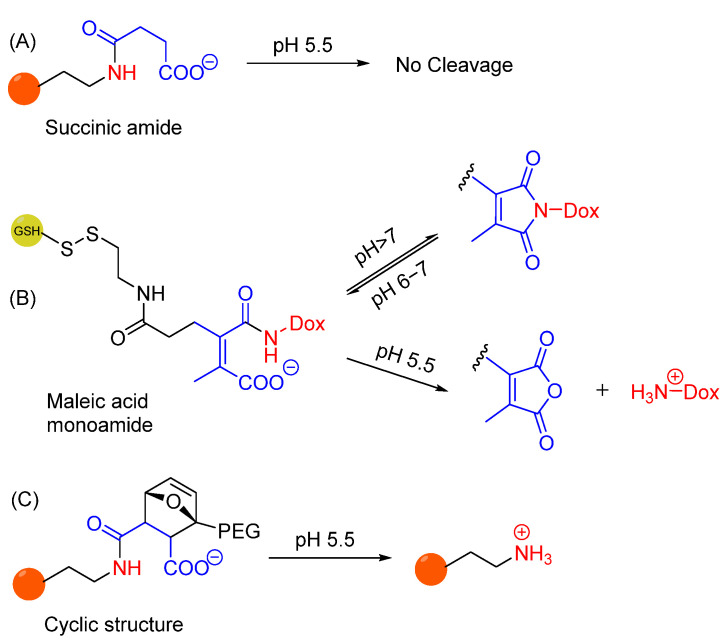
(**A**) No hydrolytic cleavage of amide bond present in succinic amide at pH 5.5. (**B**) Hydrolysis of maleic acid derivative (e.g., doxorubicin prodrug). Hydrolysis at neutral pH competes with formation of stable imide, while acidic conditions promote drug release. (**C**) Replacement of double bond in maleic acid derivative promotes amine scaffold release under acidic conditions.

Amide bonds are known to be physiologically stable and not suitable for drug release in acidic environments. The hydrolytic lability of amide bonds under acidic conditions can be improved by the presence of a carboxylate group in proximity to monoamides of maleic acid derivatives (Figure 20A,B). This structure is stable at pH 7.4, but its hydrolysis at pH 5.5 results in the overall charge switch from negatively charged carboxylate to positively charged ammonium ion. The pH sensitivity is due to the presence of a double bond that locks the proximate amide and carboxylate into conformation, which does not occur when using an amide bond formed by ring opening of succinic anhydride [264]. It has been investigated that substituents of the double bond can also affect the hydrolysis rates at different pH, where disubstituted maleic acid derivatives are found to show optimal selectivity in hydrolysis. Such derivatives have been used as an acid-cleavable linker in doxorubicin prodrug and enable stability of the conjugate at neutral pH (maintaining an equilibrium with its imide form) while selectively releasing the drug even at mildly acidic pH (Figure 20B) [265]. Recently, replacement of the double bond with a cyclic structure resulted in comparable pH sensitivity, mostly due to similar conformational restriction (Figure 20C) [266].

**Figure 21:**
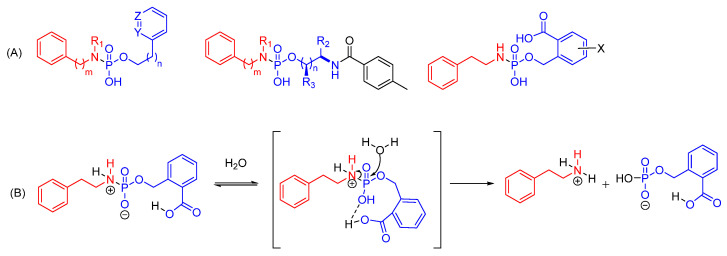
(**A**) Structures of tunable pH-sensitive phosphoramidate-based pH-sensitive linkers; (**B**) proposed mechanism for the hydrolysis of 2-carboxbenzyl phosphoramidates.

The Berkman group reported three generations of tunable pH-sensitive phosphoramidate scaffolds, claiming that proximal functional groups can promote the release of the amine payload (Figure 21A) [267,268,269]. The presence of H-bond donors (e.g., pyridinium and carboxylic acid) in the neighbouring position at the alkoxide side of phosphoramidate increases the hydrolysis rate of the P-N bond due to intramolecular H bonding (Figure 21B). It has been demonstrated that proximity of the ionizable group and pKa of both proximal ionizable group and departing amine are the main factors that can be tuned to achieve controlled release of amine payloads in acidic pH. However, the selectivity in cleavage at pH 7.4/5.5 has not been reported.

**Figure 22:**
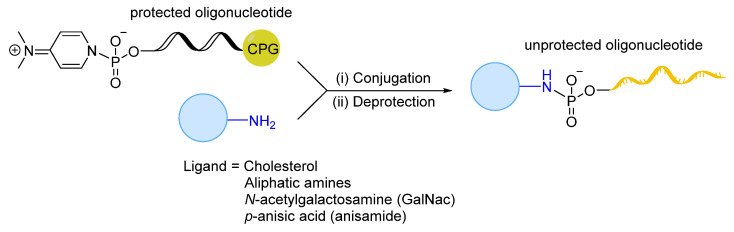
Solid-phase synthesis of oligonucleotide conjugates containing pH–sensitive phosphoramidate linkage.

Recently, a convenient solid-phase synthesis of oligonucleotide conjugates containing biodegradable, pH-sensitive phosphoramidate linkages has been reported (Figure 22) [270]. The effective release of oligonucleotides from small ligands under acidic conditions close to endosomes/lysosomes was observed while maintaining comparative stability at pH 6. The developed versatile method can potentially be used to obtain a variety of oligonucleotides (i.e., ASOs, siRNAs, miRNAs, aptamers) conjugated to different ligands, and thus could give access to compounds with increased cellular delivery and endosomal release.

To summarize, while these examples clearly serve as a basis for the development of selective and safe pH-sensitive cleavable linkers for oligonucleotide conjugates, the development of pH-sensitive linkers is an open-ended area of research, which requires more systemic evaluation and balance between stability and reactivity at different pHs.

## 8. Summary and Perspective

The impressive progress in the field of oligonucleotide therapeutics provides an opportunity to treat diseases with unmet clinical needs. Combinations of well-designed chemically modified oligonucleotides with specific conjugation or formulation led to major breakthroughs to treat multiple diseases. However, this therapeutic class is still in its early days and is set for additional advances to achieve its widespread use.

Further development of chemical modifications is required to increase the oligonucleotide therapeutic window. There is no doubt that PS modifications have been proven to be very effective, providing stability and improving ASO cellular uptake. However, the PS backbone may decrease target binding affinity and the tight PS ASO–protein interactions can lead to major toxicities. Therefore, exploring novel chemical backbones, to maintain a balance between charge and protein binding, may open the way for improving oligonucleotide drug properties. Neutral backbones such as, methylphosphonate, phosphoryl guanidine-containing (PN) backbones, amide, and triazole linkages may reduce toxicity and increase potency while maintaining oligonucleotide stability [48,271,272,273,274,275]. Furthermore, several ribose chemical modifications, such as 2′-*O*-methyl (2′-OMe), 2′-*O*-methoxy-ethyl (2′-MOE), 2′-fluoro (2′-F), locked nucleic acid (LNA), and constrained ethyl (cEt), have been employed in oligonucleotides to increase oligonucleotide stability and compensate the reduced binding affinity due to PS backbone modifications. Nevertheless, additional chemical exploration may be needed to improve further oligonucleotide activity. For example, the use of ethylene bridged nucleic acid (ENA) modification results in an improvement of binding affinity [276,277,278]. Interestingly, the use of an artificial chemical scaffold such as PMO leads to several FDA-approved ASOs to treat DMD (e.g., Viltolarsen and Casimersen). In siRNA drugs, additional chemical modifications have been explored such as glycol nucleic acid (GNA) to mitigate off-target effect, and 5′-(E)-vinyl-phosphonate (VP) for better strand selection into RISC [279,280]. These few examples demonstrate that expanding repertoire of chemical modifications with novel chemistries will advance the oligonucleotide therapeutics field.

Finding the right chemical modifications that retain oligonucleotide activity and reduce oligonucleotide toxicity is challenging. Not only chemical modification properties (e.g., lipophilicity, charge) but also their interaction with proteins (e.g., RNase H, RISC) and targets impact oligonucleotide performance, limiting the chemical space. In addition, incorporation of one type of chemical modification might not be enough to maximize oligonucleotide drug properties. A combination of multiple well-selected and position-dependent chemical modifications may be required to improve the oligonucleotide therapeutic window. However, finding the best combination needs extensive comparative studies. Therefore, an advanced chemical modification platform is needed to improve and accelerate the success rate of oligonucleotides in clinics. It is important to mention that chemical modifications that enhance ASO activity do not necessarily benefit siRNA drugs (and vice versa), adding complexity in developing oligonucleotide drugs.

Despite many advantages, chemical modifications are inadequate to improve the site-specific delivery of oligonucleotides. Various formulation and conjugation strategies are required to efficiently deliver oligonucleotides to tissues/cells of interest. The use of LNPs has revolutionized the siRNA therapeutic field as utilized in the first FDA-approved siRNA drug (Patisiran). Moreover, the success of GalNAc conjugates to efficiently deliver oligonucleotides to the liver demonstrates that the functional tissue delivery of therapeutic oligonucleotides is a foundation for any clinical exploration. As of today, the GalNAc platfom is used in four FDA-approved siRNA to treat multiple liver diseases (Givosiran, Lumasiran, Inclisiran, Vutrisiran). While both lipid nanoparticles and GalNAc conjugates are clinically validated and approved delivery strategies for liver targets, efficient delivery of oligonucleotides beyond the liver and kidneys is a fundamental obstacle preventing their clinical utility. Therefore, further development of well-designed delivery ligands (e.g., peptides, antibodies, and lipids) or nanocarriers will likely enable effective oligonucleotide delivery in extra-hepatic tissues. The continued progress and development in this area will no doubt lead to many more breakthroughs in the future.

The delivery problem is not limited to reach the tissues/cells of interest but once internalized, oligonucleotides get entrapped in the endosomal compartments. To reach the target in cytosol or nucleus, the oligonucleotide should escape from endosomes before degrading in lysosomes or recycling back via exocytosis. While chemical modifications and conjugation improve efficiency and specificity of oligonucleotide delivery, they do not necessarily influence the rate of endosomal escape. The enhanced release of PS-ASOs, lipid-conjugated PS-ASOs, or GalNAc-conjugated PS-ASOs into cytosol could be due to their higher concentration in endosomal compartments (and not an improvement of endosomal release per se), resulting from their increased intracellular uptake [28,281]. Increasing oligonucleotide hydrophobicity and modulating oligonucleotide charge could be some possible ways to enhance the endosomal escape, either due to increased uptake or higher interaction with endosomal lipid bilayer. A variety of endosomolytic agents, such as, endosomal buffering polymers, endosomolytic peptides, small molecules, ionizable lipids, and cationic liposomes, have been used to improve the endosomal release of oligonucleotides. Nevertheless, some of them are non-biodegradable and non-biocompatible, which limits their future applications in oligonucleotides delivery. In addition, it is challenging to find an optimum endosomal escape agent that induces the endosomal membrane disruption without completely rupturing the endosomes and with minimum toxic effects. Designing an endosomal escape domain that is inactive in plasma but selectively turns ‘switched on’ in mildly acidic endosomal compartments could be a useful idea in finding a potential solution to drive localized endosomal membrane disruption [282,283].

The use of reversible covalent bonds between ligand–oligonucleotide conjugates can be a promising methodology to overcome the endosomal escape barrier. Hydrolytic cleavage of pH-sensitive linkers in endosomal compartments could be one of the factors to trigger the escape of oligonucleotides from endosomes by changing their electrostatic interaction with endosomal membrane. A hydrazone linker has been commonly used as pH-sensitive linker in oligonucleotide conjugates and this area is set for supplementary advances to be more advantageous in oligonucleotide delivery. For optimum utility of pH-sensitive linkers to overcome the delivery problem of oligonucleotide conjugates, certain parameters should be followed: 1. covalently linked linkers should be inert in plasma to avoid the premature release of oligonucleotides and reduce off-target effects; 2. linkers should be selectively labile inside the endosomes to expose its endosomal escape domain; 3. the escape should not be through complete endosomal rupture but localized endosomal membrane disruption; 4. the linker should enhance the endosomal escape while maintaining the depot effect and low levels of toxicity; and 5. linkers should be easily synthesized and cost-effective, and the by-products should not be toxic at cellular and systemic level.

A combination of various chemical manipulations is required to overcome the delivery problem of oligonucleotide therapeutics. Future expansion of the chemical space will establish a path toward advancing the oligonucleotide therapeutic field.

## Figures and Tables

**Table 1:** Different chemical modifications used in oligonucleotide therapeutics.

Chemical Modification	Drug Name	Oligonucleotide Class	Indication	Advantages and *Disadvantages* of Modification
Approved Oligonucleotide Therapeutics				
PS (DNA)	VITRAVENE^®^ (fomivirsen)	ASO	CMV retinitis (1998 withdrawn)	**PS backbone modification:** - Increases nuclease resistance; - Enhances serum protein binding; - Improves cellular uptake; - Does not interfere with RNase H activity; - *May decrease target binding affinity;* - *Induces major toxicities due to protein interactions.*
2′-OMe/2′-F, PS	GIVLAARI^®^ (givosiran)	siRNA	Acute hepatic porphyria (2019)	**2′-sugar modification:** - Enhances the oligonucleotide stability; - Increases nuclease resistance; - Improves the binding affinity towards target RNA; - Reduces the immunogenicity; - *Not all sugar modifications fit all classes of oligonucleotides;* - *It does not necessarily improve the delivery of oligonucleotides.*
	OXLUMO^®^ (lumasiran)	siRNA	Primary hyperoxluria (2020)	
	LEQVIO^®^ (inclisiran)	siRNA	Heterozygous familial hypercholesterolemia (HeFH) or clinical atherosclerotic cardiovascular disease (ASCVD) (2020, EMA), (2021, FDA)	
	AMVUTTRA^®^ (vuttrisiran)	siRNA	Hereditary ATTR (hATTR) (2022)	
2′-F/2′-OMe	MACUGEN^®^ (pegaptanib)	Aptamer	Neovascular AMD (2004, withdrawn)	
2′-OMe	ONPATTRO^®^ (patisiran)	siRNA	Hereditary ATTR (hATTR) (2018)	
5′-Me-C, PS, 2′-*O*-MOE,	KYNAMRO^®^ (mipomersen)	ASO	Homozygous familial hypercholesterolemia (HoFH) (2013 withdrawn)	**5′-Me-C nucleobase modification:** - Well tolerated; - Reduces the immunostimulatory profile; - Improves oligonucleotide stability; - *Bulkier modification on nucleobase can negatively affect the oligonucleotide activity.*
	SPINRAZA^®^ (nusinersen)	SSO	Spinal muscular atrophy (SMA) (2016)	
	WAYLIVRA^®^ (volanesorsen)	ASO	Familial chlylomicronemia syndrome (FCS) (2019)	
	TEGSEDI^®^ (inotersen)	ASO	Hereditary ATTR (hATTR) (2018)	
	QALSODY^®^ (Tofersen)	ASO	Amyotrophic lateral sclerosis (ALS) (2023)	
PMO	EXONDYS 51^®^ (eteplirsen)	SSO	Duchene muscular dystrophy (DMD) (2016)	**PMO scaffold modification:** - Improves stability; - Enhances efficacy and specificity; - Increases nuclease resistance; - Increases water solubility; - Improves modulating affinity for target RNA; - *Reduces serum protein binding resulting in rapid clearance;* - *Limits the tissue distribution.*
	VYONDYS 53^®^ (golodirsen)	SSO	Duchene muscular dystrophy (DMD) (2019)	
	VILTEPSO^®^ (viltolarsen)	SSO	Duchene muscular dystrophy (DMD) (2020)	
	AMONDYS 45^®^ (casimersen)	SSO	Duchene muscular dystrophy (DMD) (2021)	
**Other chemical modifications under clinical investigation**				
PN	WVE-N531 *	SSO	Duchene muscular dystrophy (DMD) (Phase 1/2)	**PN backbone modification:** - Increases nuclease resistance.
	WVE-003 *	ASO	Huntington’s Disease (Phase 1/2)	
tcDNA	SQY51	SSO	Duchene muscular dystrophy (DMD) (Phase 1/2)	**tcDNA sugar modification:** - Increases stability of the tcDNA/RNA duplex; - Increases nuclease resistance.

* May also include modifications other than PN backbone modification.

**Table 2:** Sequences of common CPPs used in oligonucleotide delivery.

Family	Peptide	Sequence	Reference
Cationic	Tat-(43–60)	LGISYGRKKRRQRRRPPQ	[159]
	Tat-(48–60)	GRKKRRQRRRPPQ	[159]
	LAH-L1 (cationic amphipatic)	KKALLAHALHLLALLALHLAHALKKA	[150]
Amphipathic anionic	Penetratin	RQIKIWFQNRRMKWKK	[160]
	E5 (derived from HA2)	GLFEAIAEFIEGGWEGLIEGCA	[161]
	INF7 (derived from HA2)	GLFEAIEGFIENGWEGMIDGWYGC (dimer)	[162]
	GALA	WEAALAEALAEALAEHLAEALAEALEALAA	[154]
Penetration Accelerating Sequence-Hydrophobic	Cathepsin D domain	GKPILFF	[163]
	EED6	GFWFG	[164]
	EED7	GWWG	[164]

## Data Availability

No new data were created.
